# Global trends in clozapine utilisation between 2014 and 2024: a longitudinal epidemiological study with data from 75 countries

**DOI:** 10.1016/j.lanepe.2026.101602

**Published:** 2026-02-03

**Authors:** Ita Fitzgerald, Sarah O'Dwyer, Ciara Ní Dhubhlaing, Siobhan Gee, Laura J. Sahm, Amanda Wheeler, Eoin Hurley, Leena Saastamoinen, Anna Waksmundzka-Walczuk, Grainne Donohue, David Shiers, Veenu Gupta, Jo Howe, Christoph U. Correll, Mikkel Højlund

**Affiliations:** a Pharmacy Department, St Patrick's Mental Health Services, Dublin, Ireland; b Pharmaceutical Care Research Group, School of Pharmacy, University College Cork, Ireland; c Department of Medicine, St Patrick's Mental Health Services, Dublin, Ireland; d College of Mental Health Pharmacy, United Kingdom; e Pharmacy Department, South London and Maudsley NHS Foundation Trust, London, United Kingdom; f Institute of Pharmaceutical Sciences, Kings College London, United Kingdom; g Pharmacy Department, Auckland City Hospital, Auckland, New Zealand; h Centre for Mental Health, Griffith University, Brisbane, Australia; i Faculty of Health & Medical Sciences, University of Auckland, New Zealand; j Department of Psychology, Durham University, United Kingdom; k Information and Research Section, Information and Development Services, Finnish Medicines Agency (Fimea), Finland; l Healthcare Institution in Starachowice, Poland; m School of Pharmacy, College of Health and Life Sciences, Aston University, United Kingdom; n Academic Institute, St Patrick's Mental Health Services, Dublin, Ireland; o School of Medicine, University College Dublin, Dublin, Ireland; p Psychosis Research Unit, Greater Manchester Mental Health NHS Trust, Manchester, United Kingdom; q University of Manchester, Manchester, United Kingdom; r School of Medicine, Keele University, Keele, United Kingdom; s Northwell, New Hyde Park, NY, USA; t Donald and Barbara Zucker School of Medicine at Hofstra/Northwell, Department of Psychiatry and Molecular Medicine, Hempstead, NY, USA; u Department of Child and Adolescent Psychiatry, Charité - Universitätsmedizin Berlin, Berlin, Germany; v German Center for Mental Health (DZPG), Partner site Berlin, Berlin, Germany; w Einstein Center for Population Diversity (ECPD), Berlin, Germany; x Department of Psychiatry Aabenraa, Mental Health Services Region of Southern Denmark, Aabenraa, Denmark; y Department of Regional Health Research, University of Southern Denmark, Odense, Denmark

**Keywords:** Clozapine, Schizophrenia, Treatment resistant schizophrenia, Global health, Prescribing, Drug utilisation, Prescribing trends

## Abstract

**Background:**

Clozapine underutilisation in treatment-resistant schizophrenia represents a global public health challenge. We aimed to investigate contemporary trends of clozapine utilisation internationally.

**Methods:**

National estimates of clozapine utilisation were obtained for 75 countries through analysis of national prescribing databases and global pharmaceutical sales data from the IQVIA Multinational Integrated Data Analysis System. The annual national prevalence of clozapine utilisation was calculated between 2014 and 2024 via the number of defined daily doses (DDD) utilised per 1000 inhabitants per day (DDD/1000 inhabitants/day). Time trends in the annual prevalence of utilisation were tested using linear regression. Haematological monitoring stringency and the number of psychiatrists were explored as predictors of clozapine utilisation.

**Findings:**

In 2024, the global average clozapine utilisation was estimated as 0.46 DDD/1000 inhabitants/day, being greatest in New Zealand (2.99 DDD/1000 inhabitants/day), and Finland (2.61 DDD/1000 inhabitants/day) and lowest in Singapore (0.0007 DDD/1000 inhabitants/day). Over the period analysed, worldwide clozapine use increased by 0.13 DDD/1000 inhabitants/day. In 45 (60.0%) countries clozapine utilisation increased significantly, in 19 (25.3%) use remained similar, but in 11 (14.7%) use decreased significantly. Higher clozapine utilisation was associated with a higher number of psychiatrists (B = 0.11 [95% CI 0.05, 0.17]), but was unrelated to haematological monitoring stringency (B = 0.007 [95% CI −0.02, 0.03]).

**Interpretation:**

Whilst global utilisation appears increasing, substantial intercountry variation in clozapine use continues. The absence of an association between haematological monitoring stringency and rate of clozapine utilisation suggests the need for measures beyond monitoring relaxation within policy and practice to systematically increase use.

**Funding:**

Provided by St Patrick’s Mental Health Services.

**Research in context**

**Evidence before this study**

PubMed was searched without language restrictions for articles published from inception until January 15th, 2026. The search focussed on identifying research assessing intercountry differences in the prevalence of clozapine utilisation and had attempted to quantify and compare national, rather than local or within-country, rates of utilisation. The following search terms were used (((clozapine) AND ((use OR prescribing OR utilisation OR utilisation))) AND (Trend)) AND ((psychosis OR “psychotic disorder∗” OR schizo∗)). The search yielded a total of 206 articles. Articles were excluded based on eligibility according to their titles. Abstracts of the remaining articles were reviewed to identify relevant studies describing research on the prevalence of clozapine utilisation in treatment-resistant schizophrenia (TRS). The largest previous study conducted on international trends in clozapine utilisation was published in 2017 (n = 17 countries) and focussed on defining the prevalence and trends of clozapine use between 2004 and 2014. Research analysing clozapine prescribing rates in Asia was subsequently published in 2016 and studied contemporary rates of clozapine use in 15 countries. Finally, a study of trends of clozapine utilisation in Eastern Europe (n = 13 countries) between 2016 and 2021 was published in 2024. Previous studies have (i) inconsistently investigated rates of clozapine use via analysis of population-level datasets, (ii) applied various metrics when defining rates of clozapine utilisation, precluding intercountry comparisons in many cases, and (iii) largely focussed on defining use in high-income countries.

**Added value of this study**

This study investigates contemporary rates and trends of clozapine utilisation in 75 countries representing all regions of the world and over 11 years between 2014 and 2024. Whether the number of psychiatrists and the stringency of haematological monitoring within individual countries were associated with clozapine use was assessed. To the best of our knowledge, study results represent the largest and most up-to-date assessment of international trends of clozapine utilisation. Previously unknown estimates of national clozapine use are now available in many countries for the first time, including many lower- and upper-middle income countries. We have also identified countries with the highest sustained rates of clozapine use in the last decade. National rates of clozapine utilisation presented here serve as a benchmark from which individual countries can assess the effectiveness of targeted interventions aimed at increasing rates of clozapine prescribing. Furthermore, study results offer the foundation for essential future research into how country-specific healthcare systems and treatment practices influence the prescribing of clozapine. Such research is a prerequisite for developing evidence-based recommendations outlining how to effectively increase patient and prescriber engagement with clozapine treatment within healthcare systems.

**Implications of all the available evidence**

Despite some observed improvements in the rate of clozapine use globally, substantial international variation in utilisation continues to be reported. Ongoing disparities in clinical practice and timely patient access to clozapine within TRS management remain. Although the number of psychiatrists was a significant predictor of clozapine utilisation, variation in use could not be adequately explained by differences in the stringency of haematological monitoring. Measures beyond relaxation of haematological monitoring requirements are likely needed to systematically increase equitable patient access to clozapine. Aligning with results of prior research, New Zealand and Finland continue to have the highest rates of sustained clozapine utilisation. Systems of clozapine management within both countries should be the focus of future research aimed at developing targeted interventions effective in increasing rates of clozapine prescribing.

## Introduction

Clozapine underutilisation in treatment-resistant schizophrenia (TRS) represents a global public health challenge. Knowledge of how to effectively improve timely patient access remains an international research priority.^1^ Clozapine is the only approved medication in managing TRS,^2^ with meta-analytically estimated treatment-resistance rates of 37% overall in people with schizophrenia or schizoaffective disorder, 40% of people with schizophrenia or with multi-episode schizophrenia, and of up to 47% in patients with >10 years of illness duration.^3^ International guidelines recognise clozapine as the most effective treatment in managing TRS,^4^ informed by evidence demonstrating clozapine as having superior effects on reducing positive, negative, and overall symptoms, alongside relapse rates.^5^ Individuals with TRS prescribed clozapine and those who provide care to them also generally report positive experiences of clozapine treatment.^1^ However, concerns remain regarding underutilisation and the extent of delayed initiation.^2^^,^^6^ Prior analyses of international prescribing trends have demonstrated >300-fold intercountry variation in utilisation.^6^, ^7^, ^8^, ^9^ Key to increasing patient access to clozapine is to increase clinician prescribing rates. The extent of geographical variation in use suggests it is possible to develop targeted interventions to increase clinician engagement. However, key characteristics of such interventions are unknown.

Barriers identified to clozapine prescribing among clinicians relate to patients, clinicians, and healthcare institutions.^2^^,^^10^^,^^11^ Primary patient-related barriers include concerns regarding frequent haematological monitoring and patients' ability to consistently adhere to clozapine and it's monitoring requirements.^2^^,^^10^ Clinicians frequently report a lack of personal experience with prescribing clozapine and concern for serious side effects as significant barriers to their increased prescribing.^10^ Institutional barriers include the absence of co-ordinated care provision across settings and inadequate clinician access to training opportunities in clozapine prescribing and monitoring.^10^ A systems-based approach to developing interventions aimed at systematically increasing clozapine prescribing rates is increasingly called for.^1^^,^^12^

Interventions necessary to increase clozapine prescribing are likely complex interventions requiring significant behavioural change among clinicians. Required interventions are also likely to be structural interventions i.e., focus on modifying the healthcare delivery contexts.^7^ Beyond knowledge of barriers and facilitators to increased clozapine prescribing, developing a detailed understanding of the characteristics of effective behaviour change interventions, and their associated implementation settings, is also necessary.^13^^,^^14^ The first step in developing this knowledge is purposeful study of international healthcare systems with the highest rates of clozapine utilisation. Such research is a prerequisite for developing evidence-based recommendations outlining how to effectively increase patient and prescriber engagement with clozapine treatment within real-world healthcare systems. The subsequent understanding of mechanisms responsible for effectively increasing prescriber engagement with clozapine treatment therein would allow for scale-up and implementation within other settings.

Whilst prior studies have examined trends of international clozapine utilisation, these have been in relatively few countries and inconsistently using population-based datasets. National rates of utilisation in most countries are unknown, particularly outside of high-income countries.^6^, ^7^, ^8^, ^9^ The primary aim of this study was to describe the prevalence of clozapine utilisation internationally and identify countries with the highest comparative usage rates. Secondary aims included to (i) examine trends in the prevalence of clozapine utilisation between 2014 and 2024 and test for the presence of linear trend changes over this time and (ii) examine whether the prevalence of clozapine utilisation within individual countries is associated the number of psychiatrists and haematological monitoring stringency.

## Methods

To build the most globally representative map of clozapine utilisation, data from different sources were combined. Initially, prescribing and administrative databases available within individual countries containing population-level data on clozapine utilisation were accessed. Such databases are mostly limited to high-income countries with developed healthcare systems.^15^ In countries where population-level data were not available by virtue of absent appropriate databases, or due to unanswered access requests, rates of national clozapine utilisation were approximated using sales data from the healthcare analytical company IQVIA, specifically the IQVIA Multinational Integrated Data Analysis System (IQVIA MIDAS) database.^16^ In all cases, data on clozapine utilisation was requested for 2014–2024, independent of age, sex or other patient characteristics. As no patient-identifying information was obtained from any data source, ethical approval was not required. For the STrengthening the Reporting of OBservational studies in Epidemiology (STROBE) Checklist, see the Supplementary Appendix.

### Data sources

National prescribing and administrative databases were eligible when they contained data representative of ≥90% of the country's population. This requirement was necessary to account for intercountry differences in systems of longitudinal clozapine dispensing and reimbursement, including provision within hospital and community settings.^17^ Information on the national representativeness of datasets was confirmed with responsible organisations. Where national databases were available but did not meet the standard of population representativeness, rates of clozapine utilisation were approximated from the IQVIA MIDAS database to increase validity of the estimates.

IQVIA MIDAS captures global sales data of specific pharmaceutical products based on various distribution channels, including manufacturers, wholesalers, hospitals, and retail pharmacies, and applies international standardisation to allow comparisons of national sales volumes.^16^^,^^18^ The average national coverage of MIDAS data is estimated at 88%. Within countries where the MIDAS database does not have 100% market coverage, adjustments are made to estimate the total sales volume based on knowledge of the market share of participating wholesalers and retail or hospital pharmacies. Data from IQVIA MIDAS have been validated against external and alternative sources of sales data.^16^^,^^18^ IQVIA MIDAS data has been used to evaluate multinational consumption of various medicines.^19^, ^20^, ^21^, ^22^ Like in previous studies,^19^, ^20^, ^21^, ^22^ sales data were used as a proxy for clozapine utilisation. Characteristics of national databases and data contained within IQVIA MIDAS are outlined within the Supplementary Appendix, including the extent to which reported rates of clozapine utilisation reflect utilisation within different sectors (e.g., community and hospital settings) and the percentage of market coverage represented by IQVIA MIDAS data within relevant countries. The source of information informing the clozapine utilisation rates presented in the case of each country is also contained therein. To assess the potential impact of adjustments made by IQVIA in the case of incomplete market coverage, where rates of national clozapine utilisation were available within both IQVIA MIDAS and a national prescribing and administrative dataset (n = 18), the level of agreement in 2024 rates of clozapine utilisation was assessed using Spearman's rank correlation coefficient.

### Quantifying clozapine utilisation

To describe the national prevalence of clozapine utilisation within individual countries each year over the study period, the outcome metric used was the rate of clozapine utilisation, expressed as the number of defined daily doses (DDD) utilised per 1000 inhabitants per day (DDD/1000 inhabitants/day). The DDD represents the assumed average maintenance dose per day for a medicine used for its main indication in adults.^23^ The agreed DDD of clozapine is 300 mg.^24^ DDD/1000 inhabitants/day provides an approximation of the proportion of a country's population treated with a medication. The World Health Organisation (WHO) endorses the Anatomic Therapeutic Chemical and Defined Daily Dose methodology as the gold standard for drug utilisation research.^23^ Use of DDD/1000 inhabitants/day is recommended as the preferred measure of drug utilisation for medicines prescribed over extended periods to manage chronic conditions, and where there is good agreement between the average prescribed daily dose and the DDD.^23^

Annual clozapine utilisation estimated as 10 DDD/1000 inhabitants/day can be interpreted as follows: among 1000 inhabitants, 10 DDDs of clozapine are utilised on average, on any given day of the year. Alternatively, this can be expressed as 10/1000 (1%) of the population are receiving clozapine each day that year.^25^ Estimates of the global prevalence of schizophrenia typically range between 0.30–0.75%.^11^ Considering the estimate of treatment-resistance of 40% among people with schizophrenia,^3^ and although not all will be eligible to receive or respond to clozapine,^26^ a robust benchmark for the proportion of a country's population who should be prescribed clozapine at any one time is approximately 0.20% (i.e., 2 DDD/1000 inhabitants/day).

### Data analysis

The DDD/1000 inhabitants/day for each country was calculated using:^23^

$$\frac{\left(\text{Number}\;\text{of}\;\text{DDD}\;\times \;1,000\right)\;/\;\text{total}\;\text{population}}{365}$$

To account for the peak age of onset of schizophrenia being estimated as approximately 20 years,^27^ and intercountry differences in age structures, namely the population proportion of children and young people, the basis for the total population used was the number of people aged 15+ in each calendar year analysed. Population estimates were obtained from the United Nations World Population Prospects and the Population Division Data Portal.^28^ To test for time trends in the prevalence of clozapine utilisation within individual countries between 2014 and 2024, a univariate linear regression model (assuming a linear trend in clozapine utilisation), was used. One regression model was fitted per country, with year as the only independent variable.^19^, ^20^, ^21^, ^22^ The dependent variable i.e., DDD/1000 inhabitants/day of clozapine utilised, was log-transformed to better meet model assumptions, including modelling a linear relationship between clozapine utilisation and time. Natural logarithm transformation also facilitated coefficient interpretation as a relative change in clozapine utilisation over the study period. Thus, trends in the prevalence of clozapine utilisation between 2014 and 2024 were expressed as an average annual percentage change, calculated by [exp (the coefficient of the year variable)–1] × 100%.^21^^,^^22^ Residual analysis was undertaken for each linear regression model, including checks for assumptions of normality and constant variance.

To calculate worldwide rates of clozapine use in 2014 versus 2024, and stratify utilisation according to income level, individual country estimates were pooled using a DerSimonian and Laird's random-effects model. Rather than calculating an arithmetic mean, use of a random effects model was applied in pooling estimates to better account for heterogeneity in rates of clozapine utilisation reported across countries.^19^ Confidence intervals for national utilisation rates were calculated using the Poisson method. Income levels (lower-middle, upper-middle and high-income) were assigned to each country based on the 2024 World Bank income classification levels.^29^

Finally, we examined the association between clozapine utilisation in 2024 and the (1) haematological monitoring stringency, and (2) number of psychiatrists/100,000 population within individual countries. The haematological monitoring stringency was identified as a potentially important predictor of variation in utilisation given that haematological monitoring is frequently discussed as a potential barrier to increased clozapine prescribing.^2^^,^^10^^,^^11^ The number of psychiatrists nationally served as a proxy for the availability/accessibility of national mental healthcare services. Estimates of the number of psychiatrists/100,000 population were obtained from the WHO's European Health Information Gateway for countries within the European Union,^30^ and from the WHO's Mental Health Atlas for all other countries.^31^ Regarding haematological monitoring, Stringency Indices assigned to individual countries using the methodology outlined by Oloyede et al. were applied.^32^ This methodology is explained further in the Supplementary Appendix.

The relationship between these variables and clozapine utilisation was first assessed using Spearman's rank correlation coefficient. Subsequently, a multiple linear regression analysis was conducted, using log-transformed DDD/1000 inhabitants/day of clozapine as the dependent variable. Regression results are presented as the log-transformed dependent variable. Residual diagnostics, analogous to those applied in the univariate linear regression analyses, were conducted. Multicollinearity was evaluated using the Variance Inflation Factor (VIF); the resulting VIF of 1.63 suggests minimal multicollinearity. Further details of the regression model are contained within the Supplementary Appendix. 95% confidence intervals (CIs) and p-values were reported for all statistical tests with alpha = 0.05. Data analysis was performed using SPSS (version 29.0.2.0) and RStudio (version for macOS 13).

### Role of the funding source

The funding source was not involved in study design, including the collection, analysis or interpretation of data, in the writing of the manuscript or in the decision to submit for publication.

### Involvement of people with lived experience

People who have received a diagnosis of schizophrenia were involved in interpreting and presenting study results, and the preparation of this manuscript.

## Results

Rates of clozapine utilisation were obtained for 75 countries and territories internationally, representing approximately 76% of the world's population.^13^ High-income countries accounted for 61% of countries.^29^ In 25 countries, rates of clozapine utilisation were obtained from national administrative and prescribing datasets. A high level of agreement was demonstrated when rates of clozapine utilisation in 2024 obtained from IQVIA MIDAS and national administrative and prescribing datasets were compared, rho = 0.90 [0.73, 0.96]. Further details are available in the Supplementary Appendix. Rates of clozapine utilisation in 2014 and 2024, and the average annual change in use, for all countries assessed are presented in Table 1.

**Table 1:** Estimates of national clozapine utilisation in 2014 and 2024 and the average annual percentage change in utilisation. Countries are listed in descending order of clozapine utilisation rates in 2024. Rows are shaded according to income level of the country, with the lightest colour indicating high-income countries.

Country	DDD/1000 inhabitants/day		Average annual change 2014–2014	
	2014	2024	Percentage change in utilisation (95% CI)^b^	P value
New Zealand^a^	3.22	2.99	−1.10 [−1.98, −0.30]	0.02
Finland^a^	2.77	2.61	−0.60 [−1.00, −0.20]	0.01
Montenegro^a^	1.16	1.34	1.71 [0.51, 3.04]	0.01
Croatia^a^^,^^c^	0.95	1.10	1.18 [1.41, 2.12]	<0.001
Bulgaria	0.63	1.10	3.66 [2.63, 4.71]	<0.001
Serbia	0.77	1.09	2.94 [1.61, 4.29]	<0.001
Bosnia and Herzegovina	0.85	1.06	2.74 [1.91, 3.67]	<0.001
Greenland^a^	1.22	1.02	−1.49 [−4.69, 1.71]	0.32
Faroe Islands^a^^,^^d^	0.80	1.00	2.12 [0.90, 3.36]	0.005
Slovenia^a^	0.93	0.99	0.40 [0.10, 0.80]	0.01
Canada	0.83	0.98	1.00 [0.20, 1.70]	0.02
Ireland	1.04	0.94	−0.99 [−1.78, −0.10]	0.02
Australia	0.92	0.93	−0.10 [−0.60, 0.40]	0.74
Switzerland	0.91	0.89	0.10 [−0.40, 0.50]	0.81
Belarus	0.70	0.85	2.40 [1.71, 3.15]	<0.001
Estonia^a^	0.56	0.85	2.84 [1.21, 4.49]	0.03
Denmark^a^	0.60	0.81	2.02 [1.00, 2.94]	0.001
Hong Kong	0.75	0.78	0.40 [−0.20, 1.10]	0.17
Greece	0.59	0.77	2.94 [2.12, 3.66]	<0.001
United Kingdom (whole)^e^	0.70	0.71	1.00 [−0.70, 0.70]	0.92
Scotland^a^	0.88	0.80	−0.99 [−1.49, −0.50]	0.002
Wales^a^	0.01	0.02	5.86 [0.90, 10.96]	0.024
Egypt	0.16	0.72	14.57 [10.96, 18.41]	<0.001
France	0.43	0.71	5.13 [4.49, 5.76]	<0.001
Iceland^a^	0.65	0.67	−0.59 [−1.98, 0.90]	0.39
Hungary	0.60	0.64	0.60 [0.10, 1.11]	0.02
Netherlands^a^	0.55	0.63	0.90 [−0.10, 1.92]	0.07
Slovakia^a^	0.48	0.62	2.43 [2.12, 2.84]	<0.001
Latvia^a^	0.49	0.61	3.25 [1.82, 4.71]	<0.001
Poland^a^	0.50	0.60	1.51 [0.20, 2.74]	0.02
Germany^a^^,^^c^	0.59	0.60	0.50 [−0.20, 1.21]	0.13
Taiwan	0.54	0.57	0.20 [−0.60, 1.01]	0.57
Norway^a^	0.61	0.56	−0.79 [−1.10, −0.55]	<0.001
Sweden^a^	0.58	0.56	−0.50 [−0.60, −0.30]	<0.001
Austria	0.60	0.55	−0.80 [−1.30, −0.30]	0.002
Portugal^a^	0.34	0.51	3.76 [2.63, 5.02]	<0.001
Lithuania^a^	0.49	0.50	1.00 [0.10, 1.90]	0.03
Italy^a^^,^^c^	0.40	0.50	3.04 [1.1, 4.49]	<0.001
Thailand	0.30	0.49	6.18 [2.53, 10.07]	0.004
Spain^a^	0.33	0.48	3.67 [3.05, 4.20]	<0.001
Czechia	0.38	0.46	2.12 [1.10, 3.04]	<0.001
Romania	0.22	0.39	4.81 [3.26, 6.18]	<0.001
Uruguay	0.21	0.36	4.29 [2.02, 6.61]	0.002
Turkey	0.24	0.36	4.19 [2.94, 5.55]	<0.001
Lebanon	0.11	0.33	4.18 [1.92, 22.62]	0.02
Belgium^a^	0.25	0.32	2.63 [2.12, 3.25]	<0.001
United States	0.45	0.29	−4.30 [−5.26, −3.34]	<0.001
Russia	0.36	0.25	−3.05 [−4.59, −1.39]	0.002
South Africa	0.18	0.25	1.50 [−4.20, 7.46]	0.58
China	0.19	0.22	1.71 [0.50, 2.84]	0.01
Argentina	0.20	0.21	1.81 [0.60, 3.04]	0.009
Saudi Arabia	0.20	0.19	0.10 [−5.92, 6.50]	0.97
Malaysia^a^^,^^c^	0.14	0.16	5.55 [0.10, 10.85]	0.04
Tunisia	0.07	0.16	14.57 [10.96, 18.41]	<0.001
Luxembourg^a^	0.09	0.14	3.87 [1.61, 6.28]	0.004
Dominican Republic	0.07	0.14	8.76 [4.49, 13.20]	<0.001
Kazakhstan	0.10	0.13	2.92 [−0.10, 6.08]	0.06
Japan	0.02	0.11	16.90 [14.79, 19.10]	<0.001
Korea	0.20	0.11	−1.98 [−5.73, 1.92]	0.27
Puerto Rico	0.12	0.09	−2.18 [−3.54, −0.89]	0.004
Philippines	0.05	0.08	13.09 [6.82, 19.72]	<0.001
India	0.04	0.06	5.55 [3.56, 7.57]	<0.001
Brazil	0.02	0.06	10.74 [7.68, 13.88]	<0.001
Chile	0.06	0.05	−0.99 [−2.27, 0.30]	0.12
Bangladesh	0.04	0.05	1.82 [−2.08, 5.86]	0.32
Ecuador	0.02	0.03	7.89 [2.43, 12.98]	0.005
Pakistan	0.02	0.03	2.94 [−2.66, 8.98]	0.27
Sri Lanka	0.02	0.04	10.96 [1.61, 21.17]	0.02
Indonesia	0.007	0.03	10.30 [2.00, 19.72]	0.03
Venezuela	0.01	0.02	−16.14 [−40.73, 18.53]	0.28
Mexico	0.003	0.01	11.96 [6.50, 17.58]	<0.001
Vietnam	0.002	0.01	13.52 [5.65, 22.02]	0.003
Colombia	0.01	0.009	−2.67 [−11.75, 7.46]	0.55
Peru	0.009	0.007	−5.16 [−9.25, −0.89]	0.02
Jordan	0.002	0.001	28.40 [−6.67, 76.82]	0.11
United Arab Emirates	0.0002	0.001	21.12 [1.41, 44.78]	0.04
Singapore	0.13	0.0007	−28.26 [−40.19, −13.93]	0.003

Abbreviations: CI, Confidence Interval; DDD/1000 inhabitants/day, Defined Daily Dose per 1000 inhabitants per day.

The average annual change was expressed as the average annual percentage change, calculated by [exp (the coefficient of the year variable) – 1] x 100%.

a Asterix highlights countries where the rate of clozapine utilisation was obtained from national prescribing and administrative datasets. In all other cases, the estimates were obtained via IQVIA MIDAS data. Copyright IQVIA. All rights reserved.

b The average annual change in utilisation was calculated using a linear regression model, with log-transformed DDD/1000 inhabitants/day as the dependent variable and year as the only independent variable.

c In the case of these countries, 2024 estimates of clozapine utilisation were derived from 2023 figures, as rates of utilisation were not yet processed when requested initially and again in August 2025.

d Estimates of clozapine utilisation were not available for 2014 and 2015. Estimates derived from 2016 figures used as estimate for utilisation within these years.

e Data on individual countries within the United Kingdom were only available in the case of Scotland and Wales.

In 2024, overall global clozapine use was 0.46 DDD/1000 inhabitants per day, representing an increase of 0.13 DDD/1000 inhabitants/day from 2014. Table 2 provides a summary of absolute and relative changes in clozapine use between 2014 and 2024 worldwide and stratified by country income level.

**Table 2:** Worldwide pooled estimates of clozapine utilisation in 2014 and 2024 and stratified by country income level.

	Clozapine utilisation (DDD/1000 inhabitants/day)		Relative percentage increase in rate of clozapine utilisation (2014–2024)
	2014	2024	
Worldwide (n = 75)	0.33	0.46	+39.3%
High-income countries (n = 46)	0.63	0.68	+7.9%
Upper-middle income countries (n = 19)	0.23	0.33	+30.3%
Lower-middle income countries (n = 10)	0.05	0.14	+35.7%

Author analysis based on IQVIA MIDAS quarterly volume sales data for the period 2014–2024, reflecting estimates of real-world activity. Copyright IQVIA. All rights reserved.

In 45 (60.0%) of the 75 countries assessed, clozapine use increased significantly from 2014 to 2024. In 11 (14.7%) countries clozapine use decreased significantly, while in the remaining 19 countries (25.3%) clozapine use did not change significantly. Although clozapine utilisation was highest in high-income countries, the largest relative increases in use occurred in lower-middle (35.7% increase) and upper-middle (30.3%) income countries, compared to 7.9% in high-income countries. Within high-income countries, the largest relative increase in clozapine utilisation was seen in Japan with a 16.9% [14.8, 19.1] increase between 2014 and 2024. The largest decrease in utilisation rates between 2014 and 2024 was seen in Singapore (28.3% [−40.2, −13.9]).

### Countries with the highest prevalence of clozapine utilisation

**Fig. 1:**
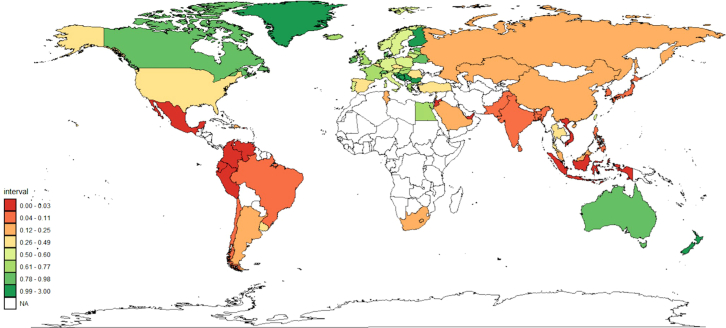
Global utilisation of clozapine in 2024. Utilisation rates are expressed in defined daily doses (DDD)/1000 inhabitants/day. NA = No data. Author analysis based on IQVIA MIDAS quarterly volume sales data for the period 2014–2024, reflecting estimates of real-world activity. Copyright IQVIA. All rights reserved.

A map representing global rates of clozapine utilisation in 2024 is provided in Fig. 1.

**Fig. 2:**
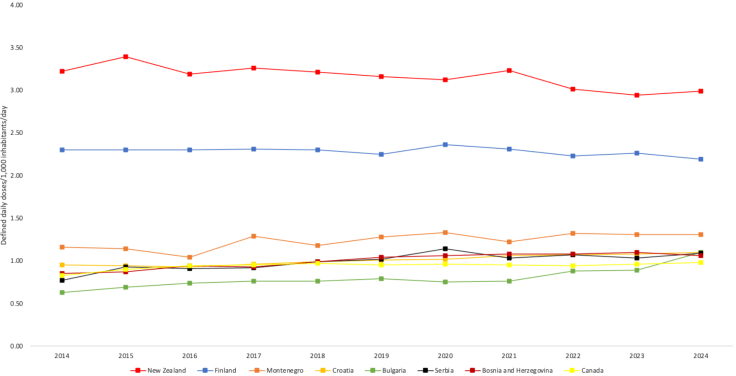
Estimates of clozapine utilisation between 2014 and 2024 in countries represented in the top tenth percentile of the dataset according to 2024 utilisation estimates. Author analysis based on IQVIA MIDAS quarterly volume sales data for the period 2014–2024, reflecting estimates of real-world activity. Copyright IQVIA. All rights reserved.

Clozapine utilisation was highest in New Zealand (2.99 DDD/1000 inhabitants/day), and Finland (2.61/1000 inhabitants/day). In 2014, clozapine utilisation was also highest in New Zealand and Finland, although there was a minor decrease in utilisation in both countries over the study period, −1.10% [95%CI = −1.98, −0.30] and −0.60 [−0.10, −0.20], respectively. Clozapine utilisation was significantly larger in both countries than in countries identified as having the third, (Montenegro, 1.34 DDD/1000 inhabitants per day), fourth (joint—Croatia and Bulgaria, 1.10 DDD/1000 inhabitants per day) and fifth (Serbia, 1.09 DDD/1000 inhabitants/day) highest rates of utilisation. Clozapine use was lowest in Singapore (0.0007 DDD/1000 inhabitants/day) and the United Arab Emirates (0.001 DDD/1000 inhabitants per day). Countries represented in the top tenth percentile of clozapine utilisation in 2024 are shown in Fig. 2. Longitudinal changes in use are also displayed.

### Clozapine utilisation and association with haematological monitoring stringency and the number of psychiatrists

**Fig. 3:**
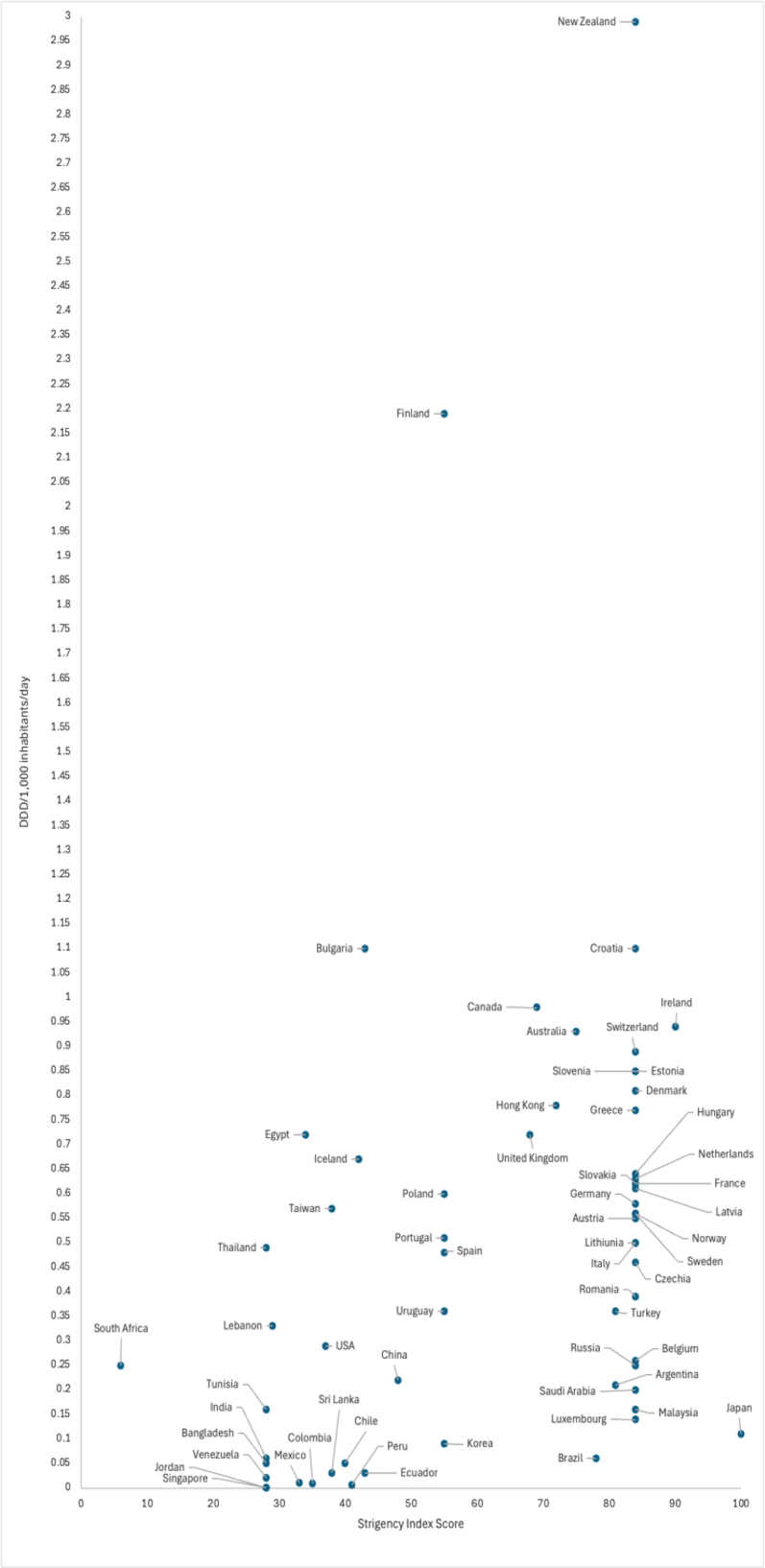
Plot of individual countries' clozapine utilisation in 2024 by assigned haematological Stringency Index.^18^ Author analysis based on IQVIA MIDAS quarterly volume sales data for the period 2014–2024, reflecting estimates of real-world activity. Copyright IQVIA. All rights reserved.

Estimates of the number of psychiatrists/1000,000 population and an assigned haematological stringency were available for 60 (80%) countries. A moderately positive correlation was seen for the number of psychiatrists/100,000 population and clozapine utilisation, rho = 0.60 [0.40, 0.74]. The relationship between the haematological monitoring stringency and clozapine use was also moderately positive, rho = 0.43 [0.19, 0.62]. Fig. 3 provides an overview of the correlation between national Stringency Indices and rates of clozapine use in 2024. A similar plot outlining the correlation between number of psychiatrists nationally and clozapine utilisation is contained in the Supplementary Appendix.

When both variables were considered within the multivariable regression model, the association between clozapine use and the number of psychiatrists remained significant (B = 0.11 [0.05, 0.17]), while the relationship with haematological monitoring stringency did not (B = 0.007 [−0.02, 0.03]).

## Discussion

This study provides information on contemporary rates and trends of clozapine utilisation in 75 countries including all regions of the world and represents to the best of our knowledge the largest, contemporary assessment of international trends of clozapine utilisation.^6^, ^7^, ^8^, ^9^ Study results offer the foundation for essential future research into how country-specific healthcare systems and treatment practices influence the prescribing of clozapine. National rates of clozapine utilisation presented here also serve as a benchmark from which individual countries can assess the effectiveness of targeted interventions aimed at increasing rates of clozapine prescribing. Whilst the global utilisation of clozapine appears largely increasing, particularly within lower-middle and upper-middle income countries, substantial intercountry variation was observed, highlighting continued divergence in clinical practice and patient access to clozapine.^6^ Furthermore, observed utilisation rates in most countries fell substantially below the estimated population proportion eligible to receive clozapine (i.e., 0.20%). In 2024 only New Zealand and Finland were estimated as having >0.20% of their population receiving clozapine. In 7/75 (9.3%) of countries, the population estimated as receiving clozapine was between 0.10–0.20%. For the remaining 66 (88%) countries, population usage rates were <0.10%.

High rates of clozapine prescribing in New Zealand and Finland were previously identified using an alternative, dose-independent metric of utilisation.^6^ Reasons for higher comparative usage rates within both countries have not been systematically assessed. Reasons are likely complex and interacting, and include factors relating to norms within clinical practice, and the design and delivery of healthcare services. Although application in practice may diverge, high rates of clozapine use within both countries do not appear to be explained by variation in licencing agreements. In New Zealand, clozapine is licenced only for the management of treatment-resistant or treatment-intolerant schizophrenia.^17^ In Finland, clozapine has an additional licenced indication for psychosis in Parkinson's disease.^6^^,^^17^ Improved access to early intervention services is worthy of consideration as a potential source of variation. However, a recent analysis of early intervention services in New Zealand identified relatively comparable standards of practice to other international systems.^33^ Other factors potentially explaining higher rates of clozapine utilisation in New Zealand include the availability of (1) assertive community treatment teams (providing medication administration support), (2) dedicated clozapine clinics, (3) culturally specific services for Māori and Pacific people, and (4) local practice guidelines relating to clozapine treatment.^34^^,^^35^

It has been proposed as possible explanation for the higher rates of clozapine prescribing in Finland that much of the empirical research supporting clozapine's superior efficacy in TRS as originating from Finland.^6^ Greater clinician willingness to adhere to evidence-based medicine has previously been suggested as an institutional facilitator of improved clozapine prescribing.^10^ Improved access to specialist resources has also been suggested as one method of effectively increasing clinician engagement with clozapine treatment.^10^ A local support network exists in Finland composed of psychiatry, cardiology and haematology specialists providing support to psychiatrists prescribing clozapine.^2^ The extent to which access to interdisciplinary expertise, and standardised schizophrenia management via multidisciplinary teams with expertise in clozapine prescribing, explains intercountry variation in utilisation should be examined within future research.^36^ This research should include defining characteristics of teams effective in increasing clozapine prescribing, and consider the benefit of inclusion of people with experience of taking clozapine, carers, and other advocates of increased use.

Significant attention has been given to reducing the frequency of haematological monitoring associated with clozapine prescribing based on evidence of a very low absolute increase in risk of serious neutropenia after two years treatment.^37^^,^^38^ In this study, although positively correlated with clozapine utilisation within univariate analysis, haematological monitoring stringency was not identified as an independent significant predictor of variation in clozapine use beyond number of psychiatrists per country. This absence of a significant association was previously demonstrated in a study of 18 countries.^32^ Although the impact on clozapine use was modest (an approximate 12% higher average DDD/1000 inhabitants/day of clozapine utilised for every additional psychiatrist/100,000 population), use of this metric as a proxy for the availability of mental health services was found to be a better independent predictor. Whilst potentially addressing an important treatment barrier among individual patients, our results demonstrate that additional measures beyond relaxation in haematological monitoring will be needed to systematically increase clozapine prescribing rates.

The impact of changes in mandatory haematological monitoring on the well-documented fear of clozapine prescribing among clinicians requires also due consideration. Known barriers to increased clozapine prescribing among clinicians extend beyond fear of blood dyscrasias and the administrative burden of haematological monitoring. Other barriers include fear of serious cardiovascular and metabolic side-effects, therapeutic nihilism associated with the prognosis of TRS, and concerns regarding incomplete patient adherence to clozapine.^10^^,^^11^^,^^39^^,^^40^ Perception of risk associated with clozapine prescribing and the potential consequences associated with serious side effects likely contributes to the well-known fear of clozapine among clinicians.^39^^,^^40^ Institutional barriers, including inadequate support for clinicians inexperienced in prescribing clozapine and fragmented service provision, are also not addressed via changes in haematological monitoring.^2^^,^^10^ Whether significant changes in the national rate of clozapine use follow local changes in haematological monitoring parameters, for example, removal of mandatory haematological monitoring by the USA's Federal Drug Agency^41^ can be assessed using rates of national clozapine utilisation provided here.

To delineate which variables to prioritise within future research attempting to explain geographical variation in clozapine use, purposeful study of countries with consistently high utilisation rates is necessary. For example, intercountry variation in clozapine availability outside of specialist settings, including community settings with dedicated resources to support clozapine management, should be considered. Some countries restrict clozapine dispensing to primarily hospital settings.^17^ In 2013 in New Zealand, attempts to increase access to clozapine were made via changes in prescribing regulations, including facilitating prescribing by non-specialist medical and nursing practitioners under a psychiatrist's supervision. Improved access was also attempted via facilitating dispensing within community pharmacies.^34^ However, similar systems of shared care and community access exist in the UK and Australia.^17^^,^^42^^,^^43^ Both countries had significantly lower rates of clozapine use compared to New Zealand.

As highlighted, study results will inform future research aimed at understanding the characteristics of effective systems of care that have achieved and sustained high rates of clozapine use. This should include studying the impact of various factors, for example systems of clozapine reimbursement, early intervention services, clinician training and cultural norms (and/or biases) on clozapine prescribing rates. Intercountry variability in smoking status, and the impact on the estimates of DDD/1000 inhabitants/day of clozapine reported here, particularly in settings with high smoking rates, should be explored also. The impact of said variables requires study using appropriate implementation science methods such that their impact on clinician prescribing behaviours, and their interaction with various contexts within real-world settings, can be made explicit. Future research should also focus on whether such systems have successfully addressed underuse of clozapine in ethnic subgroups.^44^

Study results should be considered in the context of several limitations. First, obtaining information from each country on the number of people prescribed clozapine represents the ideal metric for calculating the prevalence of clozapine prescribing nationally. However, limiting inclusion of countries to only those where nationally representative datasets are available on the population proportion prescribed clozapine would have substantially reduced the number of countries that could be included and limited analysis to primarily high-income countries. Measuring clozapine utilisation via DDD/1000 inhabitants/day provided a fixed unit of measurement independent of cost, package size, number of prescriptions, or product strength, is the metric by which countries typically quantify and compare rates of medicines utilisation and represents the WHO-endorsed standard in drug utilisation research when comparing medicines consumption at an international level.^23^ Use of our methodology has allowed previously unknown rates of clozapine utilisation to become available for the first time, including many lower- and upper-middle income countries. However, it should be acknowledged that this metric provides an estimate of medicines utilisation, not an exact picture of use.^23^

Second, clozapine's agreed DDD of 300 mg is based on research highlighting that in most patients a therapeutic dosing range is achieved at doses between 200 and 450 mg/day.^24^^,^^45^ However, the dose required to achieve therapeutic levels varies and is impacted by several variables, for example, gender, weight and smoking status.^2^ Use of DDD/1000 inhabitants/day to quantify rates of clozapine utilisation may not align well with actual rates of clozapine utilisation in countries where the prescribed daily dose varies substantially to the DDD. Whilst the ranking of individual countries may change via use of alternative utilisation metrics, clozapine use being highest in New Zealand and Finland was previously identified using the number of people prescribed clozapine per 100,000 population as the utilisation metric.^6^ Third, despite adjustments made within the IQVIA MIDAS dataset to project total utilisation, rates of clozapine utilisation may be underestimated in countries without 100% market coverage.^16^^,^^18^ We are, however, reassured by the average market coverage being circa 90% and the high level of agreement found here between rates obtained from IQVIA MIDAS and those within national prescribing and administrative datasets.

Fourth, this study focussed on identifying the prevalence of clozapine prescribing within TRS management. Within a small number of countries clozapine is licenced for additional indications, including psychosis in Parkinson's disease and less frequently for recurrent suicidal behaviour among people with schizophrenia or schizoaffective disorder.^17^ Some prior research is available examining intercountry variation in clozapine's licencing agreements.^17^ Clozapine may also be prescribed for unlicensed indications. Thus, the ranking of individual countries may be impacted in settings where use for alternative indications is substantial. To the best of our knowledge there is no published research assessing international variation in prescribing of clozapine according to indication that would allow us to assess the impact on rankings reported here. However, the limited number of countries in which clozapine is licenced for these indications and doses of clozapine recommended in the treatment of psychosis associated in Parkinson's disease (typically 12.5–50 mg/day), and the mean dose studied in the context of suicidal ideation among people with schizophrenia or schizoaffective disorder (approximately 300 mg),^46^ both limit the impact of prescribing for alternative indications of national rates of clozapine utilisation.

Finally, in ten of the national prescribing and administrative datasets used, clozapine utilisation did not reflect prescriptions dispensed within hospital settings. However, as clozapine is prescribed longitudinally and primarily within outpatient settings,^17^ we anticipate any impact on study results to be minimal. Finally, while numerous variables could explain between-country variation in clozapine utilisation, we only examined two that could be obtained reliably for most countries, i.e., number of psychiatrists and stringency of haematological monitoring. One of the primary objectives of this study was to—in examining international trends in clozapine utilisation—facilitate future, targeted research examining systems of care effective in achieving high rates of clozapine prescribing. Such research will include expanding the number of variables that explain the enduring intercountry variation in clozapine utilisation, including the impact of structural determinants. This research should also facilitate country-specific analyses that were not possible here. While the assumption of a linear trend was consistent with previous multinational drug-utilisation studies,^20^^,^^21^ non-linear or segmented patterns may exist in some countries. Future analyses using flexible models (e.g., interrupted or piecewise regression) could more precisely capture such inflection points.

### Conclusion

Whilst global clozapine utilisation appears increasing in the past decade, this is not uniformly and demonstrated increases were modest. Substantial and clinically meaningful intercountry variation in use was seen, reflecting ongoing divergence in TRS management and much needed timely patient access to clozapine. Whilst absolute clozapine utilisation rates were highest in high-income countries, the largest relative increases in use were seen in low-middle and upper-middle income countries. In most countries’ observed rates of clozapine utilisation fell significantly below the approximate population proportion with TRS. Whilst the number of psychiatrists was an independent predictor of higher clozapine use, the haematological monitoring stringency was not. This finding supports the need for additional measures beyond changes in mandatory haematological monitoring to systematically increase clozapine prescribing rates. Clozapine utilisation has consistently been demonstrated as being highest in New Zealand and Finland. Systems of clozapine management within both countries should be the focus of future research aimed at developing targeted interventions effective in increasing patient and prescriber engagement with clozapine treatment in other countries.

## Contributors

IF conceptualised the study. IF, SoD, CNiD, and GD were responsible for funding acquisition. The study design was informed by IF, SoD, CNiD, and MH. IF and MH had access to datasets in their raw form. IF conducted the initial analysis which was verified by MH. Both IF and MH are responsible for data analysis and presentation. All authors had access to aggregate figures and were involved in interpretation and presentation of study results. IF wrote the original draft of this manuscript that was subsequently critically reviewed by all authors during draft revisions. All authors have approved the submission of the manuscript. IF had final responsibility for the decision to submit for publication.

## Data sharing statement

In the case of national administrative datasets, such datasets were publicly available or were accessed following submission of a written data access request to the data custodian within the appropriate organisation. The statements, findings, conclusions, views, and opinions contained and expressed in this article are based in part on data obtained under licence from the following: IQVIA MIDAS® sales data for the period 2014–2024: quarterly-country level sales of clozapine (N05AH02). Geography: Global Countries: Argentina, Austria, Australia, Bangladesh, Belarus, Bosnia, Brazil, Bulgaria, Canada, Chile, Chine, Colombia, Czech Republic, Dominican Republic, Ecuador, Egypt, France, Greece, Hungary, Hong Kong, India, Indonesia, Ireland, Japan, Jordan, Kazakhstan, Korea, Lebanon, Mexico, Pakistan, Peru, Philippines, Puerto Rico, Romania, Russia, Saudi Arabia, Serbia, Singapore, South Africa, Sri Lanka, Switzerland, Taiwan, Thailand, Tunisia, Turkey, UK, United Arab Emirates, Uruguay, USA, Venezuela, Vietnam. The statements, findings, conclusions, views, and opinions contained and expressed herein are not necessarily those of IQVIA. The terms of our agreement do not permit disclosure, sublicensing, or sharing of IQVIA MIDAS data. IQVIA will honour legitimate requests or MIDAS data from qualified researchers. Please contact IQVIA to seek approval for data access; a licence fee may be applied.

## Editor note

The Lancet Group takes a neutral position with respect to territorial claims in published maps and institutional affiliations.

## Declaration of interests

MH has received honoraria for lecturing/consultancy from H. Lundbeck and Otsuka. IF has received honoraria for consultancy from Teva. CUC has been a consultant and/or advisor to or has received honoraria from: AbbVie, Alkermes, Allergan, Angelini, Aristo, Autobahn, Boehringer-Ingelheim, Bristol-Meyers Squibb, Cardio Diagnostics, Cerevel, CNX Therapeutics, Compass Pathways, Darnitsa, Delpor, Denovo, Draig, Eli Lilly, Eumentis Therapeutics, Gedeon Richter, GH, Hikma, Holmusk, IntraCellular Therapies, Jamjoom Pharma, Janssen/J&J, Karuna, LB Pharma, Lundbeck, MedInCell, MedLink Global, Merck, Mindpax, Mitsubishi Tanabe Pharma, Maplight, Mylan, Neumora Therapeutics, Neuraxpharm, Neurocrine, Neurelis, Neurosterix, NeuShen, Neusignal Therapeutics, Newron, Noven, Novo Nordisk, Orion Pharma, Otsuka, PPD Biotech, Recognify Life Science, Recordati, Relmada, Response Pharmaeutical, Reviva, Rovi, Saladax, Sanofi, Seqirus, Servier, Sumitomo Pharma America, Sunovion, Sun Pharma, Supernus, Tabuk, Takeda, Teva, Terran, Tolmar, Vertex, Viatris, and Xenon Pharmaceuticals. He provided expert testimony for Janssen, Lundbeck, Neurocrine, and Otsuka. He served on a Data Safety Monitoring Board for Compass Pathways, IntraCellular Therapies, Relmada, Reviva, Rovi. He has received grant support from Boehringer-Ingelheim, Janssen and Takeda. He received royalties from UpToDate and is also a stock option or stock holder of Cardio Diagnostics, Kuleon Biosciences, LB Pharma, MedLink Global, Mindpax, Quantic, Terran. DS is an expert advisor to the NICE Centre for Guidelines; views are the authors and not those of NICE. VG is supported by Wellcome Trust (WT226798/Z/22/Z). The remaining authors have nothing to declare.
