# Supplementary material for: Global trends in clozapine utilisation between 2014 and 2024: a longitudinal epidemiological study with data from 75 countries
Source: Lancet Reg Health Eur. 2026 Feb 3;63:101602. doi: 10.1016/j.lanepe.2026.101602 (PMC12890874; doi:10.1016/j.lanepe.2026.101602)
Supplement: Supplementary Material [file mmc1.docx]

**Global trends in clozapine utilisation between 2014-2024: a longitudinal epidemiological study with data from 75 countries (supplementary appendix)**

Ita Fitzgerald, Sarah O’Dwyer, Ciara Ní Dhubhlaing, Siobhan Gee, Laura J. Sahm, Amanda Wheeler, Eoin Hurley, Leena Saastamoinen, Anna Waksmundzka-Walczuk, Grainne Donohue, David Shiers, Veenu Gupta, Jo Howe, Christoph U Correll, Mikkel Højlund

Pharmacy Department, St Patrick’s Mental Health Services, Dublin, Ireland (I Fitzgerald PhD, C Ní Dhubhlaing MSc, LJ Sahm PhD)

Pharmaceutical Care Research Group, School of Pharmacy, University College Cork, Ireland (I Fitzgerald PhD, C Ní Dhubhlaing MSc, LJ Sahm PhD)

Department of Medicine, St Patrick’s Mental Health Services, Dublin, Ireland (S O’Dwyer MD)

College of Mental Health Pharmacy, United Kingdom (C Ní Dhubhlaing MSc, Ita Fitzgerald PhD, S Gee PhD)

Pharmacy Department, South London and Maudsley NHS Foundation Trust, London, United Kingdom (S Gee PhD)

Institute of Pharmaceutical Sciences, Kings College London, United Kingdom (S Gee PhD)

Pharmacy Department, Auckland City Hospital, Auckland, New Zealand (E Hurley PhD)

Centre for Mental Health, Griffith University, Brisbane Australia ((AJ Wheeler PhD)

Faculty of Health & Medical Sciences, University of Auckland New Zealand (AJ Wheeler PhD)

Department of Psychology, Durham University, United Kingdom (V Gupta, PhD)

Information and Research Section, Information and Development Services, Finnish Medicines Agency (Fimea) (L Saastamoinen PhD)

Healthcare Institution in Starachowice, Poland (AnnaWaksmundzka-Walczuk MPharm)

School of Pharmacy, College of Health and Life Sciences, Aston University, United Kingdom (J Howe PhD, I Fitzgerald PhD)

Academic Institute, St Patrick’s Mental Health Services, Dublin, Ireland (G Donohue PhD)

School of Medicine, University College Dublin, Dublin, Ireland (G Donohue PhD)

Psychosis Research Unit, Greater Manchester Mental Health NHS Trust, Manchester, United Kingdom (D Shiers OBE)

University of Manchester, Manchester, United Kingdom (D Shiers, OBE)

School of Medicine, Keele University, Keele, United Kingdom (D Shiers, OBE)

Northwell, New Hyde Park, NY, USA (CU Correll, MD)

Donald and Barbara Zucker School of Medicine at Hofstra/Northwell, Department of Psychiatry and Molecular Medicine, Hempstead, NY, USA (CU Correll, MD)

Charité - Universitätsmedizin Berlin, Department of Child and Adolescent Psychiatry, Berlin, Germany (CU Correll, MD)

German Center for Mental Health (DZPG), partner site Berlin, Berlin, Germany (CU Correll, MD)

Einstein Center for Population Diversity (ECPD), Berlin, Germany (CU Correll, MD)

Department of Psychiatry Aabenraa, Mental Health Services Region of Southern Denmark, Aabenraa, Denmark (M Højlund PhD)

Department of Regional Health Research, University of Southern Denmark, Odense, Denmark (M Højlund PhD)

Table of Contents

[Table 1 – Characteristics of national prescribing and administrative datasets 3](#_Toc211946251)

[Table 2 - List of participating countries/regions in the IQVIA-MIDAS dataset and sector coverage 11](#_Toc211946252)

[Table 3 – Rates of clozapine utilisation in DDD/1,000 inhabitants/day across all countries (2014-2024) 14](#_Toc211946253)

[Table 4 – Comparing rates of clozapine utilisation in 2024 in countries where estimates were available in both IQVIA-MIDAS and national prescribing and administrative databases. 18](#_Toc211946254)

[Table 5 – Completed STrengthening the Reporting of OBservational studies in Epidemiology (STROBE) Checklist 20](#_Toc211946255)

[Table 6 – Details of correlation and regression analyses of factors associated with estimates of clozapine utilisation in 2024 23](#_Toc211946256)

[Plot 1 - Plot of individual countries’ clozapine utilisation in 2024 by number of psychiatrists/100,000 population 26](#_Toc211946257)

[Haematological Monitoring Stringency Index – Details of Eloyede et al method 27](#_Toc211946258)

# Table 1 – Characteristics of national prescribing and administrative datasets

| Country | Database | Number of years included in the study | Reflects clozapine consumption in community and hospital settings (Yes/No) |
| --- | --- | --- | --- |
| Belgium | Pharmanet | 2014-2024 | No  Database contains data on reimbursed medicines that have been dispensed in public pharmacies. Data on non-reimbursed medicines that public pharmacies dispense or medicines that hospital pharmacies dispense do not therefore appear in Pharmanet. |
| Croatia | HALMED, Croatian Agency for Medicinal Products and Medical Devices.  The results in the report are obtained from pharmacies, hospital pharmacies and specialist stores are very similar to the results obtained from wholesalers in the reported values. | 2014-2023 | Yes  The results in the report are obtained from pharmacies, hospital pharmacies and specialist stores are very similar to the results obtained from wholesalers in the reported values. |
| Denmark | Danish Medicines Statistics Register | 2014-2024 | Yes  Database contains data from the hospital and primary care settings. |
| Estonia | Statistical Yearbook of the State Agency of Medicines | 2014-2024 | Yes  The data provided on drug consumption is based on wholesalers’ sales to hospital pharmacies and general pharmacies. |
| Faroe Island | National Pharmacy Service | 2016-2024  *Estimates of clozapine utilization were not available for 2014 and 2015. Estimates derived from 2016 figures used as estimate | Yes  Covers hospital and community prescription. |
| Finland | FIMEA Finnish Medicines Agency | 2014-2024 | Yes  Sales figures within the database are based on the amount of medicines sold by drug wholesalers to pharmacies and hospitals. |
| Germany | AOK Research Institute (WIdO) - PharMaAnalyst | 2014-2023 | No  Evaluates prescription data for outpatient medications prescribing to the approximately 90% of the German population with statutory health insurance. |
| Greenland | Nordic Health and Welfare Statistics | 2014-2024 | Yes.  Data represent prescriptions dispensed in all healthcare settings including community and hospital settings. |
| Iceland | The Icelandic Medicines Agency and the Prescription Medicines Register | 2014-2024 | Yes  Prescription Medicines Register which contains information on all drugs dispensed in pharmacies and to individuals residing in nursing homes. |
| Italy | The Italian Medicines Agency and the Medicines Utilization Monitoring Centre – National Report on medicines use in Italy. | 2014-2023 | Yes  Reports on pharmaceutical prescription data in inpatient and outpatient settings and covers prescription items covered by the National Health System and those obtained through private purchase. |
| Latvia | The State Agency of Medicines | 2014-2024 | Yes  The Agency collates figures based on monthly sales of pharmaceutical products provided by wholesalers to all end users including community and hospital pharmacies, medical institutions and healthcare professionals. |
| Lithuania | Lithuanian State Medicines Control Agency (SMCA) | 2014-2024 | Yes  The Agency produces reports on Report on medicines consumed based on monthly sales data reported by wholesalers by law and reflects distribution across all pharmacies. |
| Luxembourg | Ministry of Health And Social Security | 2014-2024 | No  The database contains data on medicines obtained via prescription from public pharmacies and represents individuals insured under Luxembourg’s health and maternity insurance and resident in Luxembourg, which represents approximately 90% of the total resident population. |
| Malaysia | Ministry of Health Malaysia – Malaysian Statistics on Medicines Reports | 2014-2023 | Yes  Reports on total medicines consumption in the country in both hospital and community settings |
| Montenegro | Institute for Medicines and Medical Devices of Montenegro | 2014-2024 | Yes  Data kept by the Institute is based on sales of all medicines by wholesalers in Montenegro and represent both the private and public sector. |
| New Zealand | The Pharmaceutical Collection | 2014-2024 | No  The database collects drug consumption data on community-dispensed pharmaceuticals that are publicly funded. It does not collect data on prescriptions provided in hospital settings. |
| Norway | The Medicines Register at the Norwegian Institute of Public Health. | 2014-2024 | No  The Medicines Register contains information on medicines dispensed on prescription and requisition from pharmacies to humans and animals from 2004 onwards. Medicine use at the individual level in institutions (e.g. hospitals and nursing homes) is also not included in the register. |
| Poland | National Health Fund of Poland (NFZ) | 2014-2024 | Yes  Figures represent refundable prescriptions and include both public and private prescriptions.  Approximately 90% of individuals in Poland have access to the national health system. |
| Portugal | Ministry of Health - National Authority of Medicines and Health Products | 2014-2024 | No  The data provided refer to prescribed and reimbursed medicines dispensed in community pharmacies in mainland Portugal and does not represent hospital prescriptions. |
| Scotland | Public Health Scotland | 2014-2024 | No  The data provided represent medicines prescribed and/or dispensed in Scotland and does not cover prescriptions in hospitals. |
| Slovakia | National Health Information Centre | 2014-2024 | Yes  The data represent prescriptions reimbursed by health insurance companies in Slovakia, including both hospital and community settings and is representative of 100% of the population |
| Slovenia | Health Insurance Institute of Slovenia | 2014-2024 | No  Data do not include drugs dispensed in hospitals but do include drugs prescribed to hospital patients at discharge, to be collected in a community pharmacy. Data do include drugs dispensed in long-term care facilities. |
| Spain | Spanish National Health System | 2014-2024 | No  The data provided corresponds to all the consumption of official medical prescriptions receipts issued by the Spanish National Health System and dispensed in community pharmacies charged to the public funds |
| Sweden | The National Board of Health and Welfare, Statistical database on pharmaceuticals | 2014-2024 | No  The statistical database contains information on prescribed drugs dispensed at pharmacies. The database does not include medicines dispensed in hospital settings. |
| The Netherlands | Drug Information System of the Health Care Insurance Board and the Medicines and Resource Information Project (GIP) databank. | 2014-2024 | No  Database contains information on prescribe medicines based on health insurance claims based on prescriptions dispensed by pharmacies, dispensing general practitioners or medical aid suppliers.  Hospital prescriptions, administered within the hospital setting, are not included. |
| Wales | NHS Wales Shared Services Partnership | 2014-2024 | Yes  Reports on total medicines consumption in the country in both hospital and community settings. |

# Table 2 - List of participating countries/regions in the IQVIA-MIDAS dataset and sector coverage

| **Country** | **Sectors covered** | **Percentage of market coverage** |
| --- | --- | --- |
| Argentina | Retail | 82% |
| Australia | Retail and hospital | 97% |
| Austria | Retail and hospital | 100% |
| Belarus | Retail and hospital | 100% |
| Belgium | Retail and hospital | 100% |
| Bosnia and Herzegovina | Retail and hospital | 95% |
| Brazil | Retail and others (non-specified) | 97% |
| Bulgaria | Retail and hospital | 98% |
| Canada | Retail and hospital | 100% |
| Chile | Retail | 71% |
| Colombia | Retail | 37% |
| Croatia | Retail and hospital | 98% |
| Czechia | Retail and hospital | 95% |
| Denmark | Retail and hospital | 100% |
| Ecuador | Retail and others (public institutions) | 95% |
| Egypt | Retail, hospital and others (e.g., tenders, army) | 100% |
| Greece | Retail | 79% |
| Hungary | Retail and hospital | 100% |
| India | Retail, hospital and dispensing doctor | 95% |
| Ireland | Retail and hospital | 100% |
| Japan | Retail and hospital | 100% |
| Kazakhstan | Retail and hospital | 100% |
| Lebanon | Retail and others (private hospitals, institutions) | 77% |
| Lithuania | Retail and hospital | 99% |
| Luxembourg | Retail | 98% |
| Mainland China | Retail and hospital | 71% |
| Mexico | Retail and others (private hospitals, government institutions) | 100% |
| Morocco | Retail and hospital | 88% |
| Pakistan | Retail, hospital and others (non-specified) | 85% |
| Peru | Retail and others (government and private institutions) | 67% |
| Philippines | Retail and hospital | 100% |
| Poland | Retail and hospital | 100% |
| Portugal | Retail and hospital | 100% |
| Puerto Rico | Retail and hospital | Not available |
| Republic of Korea | Retails, hospitals and others (clinics) | 99% |
| Romania | Retail and hospital | 100% |
| Russia | Retail and hospital | 98% |
| Saudi Arabia | Retail, hospital and others (government institutions) | 100% |
| Serbia | Retail, hospital and others (nursing homes, private clinics) | 93% |
| Slovakia | Retail, hospital and others (deliveries to the army) | 97% |
| Slovenia | Retail, hospital and others (private doctors, public health centres, nursing homes) | 98% |
| South Africa | Retail, hospital and others (private doctors) | 100% |
| Spain | Retail, hospital and others (military, government) | 99% |
| Sweden | Retail and hospital | 100% |
| Switzerland | Retail, hospital and others (Dispensing doctors) | 100% |
| Taiwan | Retail, hospital and others  (Dispensing doctors) | 97% |
| Thailand | Retail, hospital and others (private health centres and clinics) | 93% |
| Tunisia | Retail and hospital | 100% |
| Turkey | Retail and hospital | 100% |
| Ukraine | Retail and hospital | 100% |
| United Arab Emirates | Retail and hospital | 90% |
| United Kingdom | Retail and hospital | 93% |
| United States | Retail, hospital and others (Mail order) | 86% |
| Uruguay | Retail and others (private institutions, public institutions) | 69% |
| Venezuela (Bolivarian Republic of) | Retail, hospital and others | 78% |

# Table 3 – Rates of clozapine utilisation in DDD/1,000 inhabitants/day across all countries (2014-2024)

| **Country** | **2014 DDD_1000 inhabitants_per day** | **2015 DDD_1000 inhabitants_per day** | **2016 DDD**  **_1000 inhabitants_per day** | | **2017 DDD_1000 inhabitants_per day** | | **2018 DDD_1000 inhabitants_per day** | **2019 DDD_1000 inhabitants_per day** | | **2020 DDD_1000 inhabitants_per day** | | **2021 DDD_1000 inhabitants_per day** | **2022 DDD_1000 inhabitants_per day** | **2023 DDD_1000 inhabitants_per day** | **2024 DDD**  **_1000 inhabitants_per day** |  |  |
| --- | --- | --- | --- | --- | --- | --- | --- | --- | --- | --- | --- | --- | --- | --- | --- | --- | --- |
| ARGENTINA | 0.20 | 0.18 | 0.18 | 0.18 | | 0.19 | | 0.19 | 0.20 | | 0.21 | | 0.23 | 0.21 | 0.21 |  |  |
| AUSTRALIA | 0.92 | 0.95 | 0.99 | 0.98 | | 0.98 | | 0.98 | 0.98 | | 0.97 | | 0.95 | 0.95 | 0.93 |  |  |
| AUSTRIA | 0.60 | 0.59 | 0.60 | 0.60 | | 0.61 | | 0.60 | 0.60 | | 0.56 | | 0.57 | 0.56 | 0.55 |  |  |
| BANGLADESH | 0.04 | 0.04 | 0.06 | 0.06 | | 0.07 | | 0.06 | 0.07 | | 0.05 | | 0.05 | 0.06 | 0.05 |  |  |
| BELARUS | 0.70 | 0.66 | 0.73 | 0.69 | | 0.76 | | 0.72 | 0.77 | | 0.78 | | 0.81 | 0.86 | 0.85 |  |  |
| BELGIUM | 0.25 | 0.26 | 0.27 | 0.28 | | 0.29 | | 0.30 | 0.31 | | 0.32 | | 0.32 | 0.32 | 0.32 |  |  |
| BOSNIA | 0.85 | 0.87 | 0.94 | 0.93 | | 0.99 | | 1.04 | 1.06 | | 1.08 | | 1.08 | 1.10 | 1.06 |  |  |
| BRAZIL | 0.02 | 0.02 | 0.03 | 0.02 | | 0.03 | | 0.03 | 0.04 | | 0.05 | | 0.05 | 0.05 | 0.06 |  |  |
| BULGARIA | 0.63 | 0.69 | 0.74 | 0.76 | | 0.76 | | 0.79 | 0.75 | | 0.76 | | 0.88 | 0.89 | 1.10 |  |  |
| CANADA | 0.83 | 0.90 | 0.94 | 0.95 | | 0.97 | | 0.95 | 0.96 | | 0.95 | | 0.94 | 0.96 | 0.98 |  |  |
| CHILE | 0.06 | 0.06 | 0.05 | 0.05 | | 0.05 | | 0.05 | 0.05 | | 0.05 | | 0.05 | 0.05 | 0.05 |  |  |
| CHINA | 0.19 | 0.20 | 0.19 | 0.19 | | 0.20 | | 0.22 | 0.22 | | 0.22 | | 0.21 | 0.21 | 0.22 |  |  |
| COLOMBIA | 0.01 | 0.02 | 0.005 | 0.008 | | 0.007 | | 0.007 | 0.02 | | 0.007 | | 0.007 | 0.007 | 0.009 |  |  |
| CROATIA | 0.95 | 0.94 | 0.93 | 0.96 | | 0.99 | | 1.01 | 1.02 | | 1.06 | | 1.07 | 1.08 | 1.10 |  |  |
| CZECH REPUBLIC | 0.38 | 0.38 | 0.40 | 0.42 | | 0.44 | | 0.45 | 0.46 | | 0.46 | | 0.45 | 0.44 | 0.46 |  |  |
| DENMARK | 0.60 | 0.60 | 0.60 | 0.60 | | 0.60 | | 0.60 | 0.60 | | 0.70 | | 0.70 | 0.70 | 0.70 |  |  |
| DOMINICAN REPUBLIC | 0.07 | 0.05 | 0.07 | 0.07 | | 0.06 | | 0.09 | 0.10 | | 0.08 | | 0.09 | 0.11 | 0.14 |  |  |
| ECUADOR | 0.02 | 0.02 | 0.02 | 0.03 | | 0.02 | | 0.02 | 0.02 | | 0.03 | | 0.02 | 0.03 | 0.03 |  |  |
| EGYPT | 0.16 | 0.18 | 0.22 | 0.22 | | 0.31 | | 0.42 | 0.42 | | 0.45 | | 0.44 | 0.42 | 0.72 |  |  |
| ESTONIA | 0.56 | 0.57 | 0.58 | 0.62 | | 0.64 | | 0.63 | 0.68 | | 0.67 | | 0.69 | 0.61 | 0.85 |  |  |
| FAROE ISLAND | 0.80 | 0.80 | 0.80 | 0.90 | | 0.90 | | 0.90 | 0.90 | | 0.90 | | 0.90 | 0.90 | 1.00 |  |  |
| FINLAND | 2.77 | 2.78 | 2.78 | 2.77 | | 2.75 | | 2.68 | 2.82 | | 2.75 | | 2.63 | 2.67 | 2.61 |  |  |
| FRANCE | 0.43 | 0.46 | 0.48 | 0.51 | | 0.54 | | 0.59 | 0.62 | | 0.64 | | 0.64 | 0.67 | 0.71 |  |  |
| GERMANY | 0.59 | 9.57 | 0.58 | 0.60 | | 0.61 | | 0.64 | 0.63 | | 0.60 | | 0.60 | 0.60 | 0.60 |  |  |
| GREENLAND | 1.22 | 1.34 | 1.16 | 1.45 | | 1.30 | | 1.54 | 1.59 | | 1.17 | | 1.40 | 1.01 | 1.02 |  |  |
| GREECE | 0.59 | 0.59 | 0.66 | 0.68 | | 0.68 | | 0.67 | 0.73 | | 0.73 | | 0.75 | 0.76 | 0.77 |  |  |
| HONG KONG | 0.75 | 0.73 | 0.72 | 0.71 | | 0.79 | | 0.75 | 0.77 | | 0.74 | | 0.73 | 0.74 | 0.78 |  |  |
| HUNGARY | 0.60 | 0.61 | 0.6 | 0.63 | | 0.62 | | 0.63 | 0.65 | | 0.63 | | 0.63 | 0.63 | 0.64 |  |  |
| ICELAND | 0.65 | 0.71 | 0.70 | 0.70 | | 0.71 | | 0.71 | 0.74 | | 0.71 | | 0.58 | 0.66 | 0.67 |  |  |
| INDONESIA | 0.007 | 0.02 | 0.02 | 0.03 | | 0.02 | | 0.04 | 0.04 | | 0.03 | | 0.03 | 0.03 | 0.03 |  |  |
| INDIA | 0.04 | 0.05 | 0.05 | 0.05 | | 0.06 | | 0.06 | 0.06 | | 0.06 | | 0.06 | 0.06 | 0.06 |  |  |
| IRELAND | 1.04 | 1.08 | 1.07 | 1.09 | | 1.10 | | 1.10 | 1.08 | | 1.01 | | 0.99 | 0.96 | 0.94 |  |  |
| ITALY | 0.40 | 0.40 | 0.40 | 0.40 | | 0.40 | | 0.40 | 0.50 | | 0.50 | | 0.50 | 0.50 | 0.50 |  |  |
| JAPAN | 0.02 | 0.03 | 0.03 | 0.04 | | 0.05 | | 0.05 | 0.06 | | 0.07 | | 0.08 | 0.09 | 0.11 |  |  |
| JORDAN | 0.00 | 0.0004 | 0 | 0 | | 0.001 | | 0.002 | 0.007 | | 0.008 | | 0.02 | 0.008 | 0.001 |  |  |
| KAZAKHSTAN | 0.10 | 0.12 | 0.12 | 0.14 | | 0.16 | | 0.16 | 0.15 | | 0.15 | | 0.18 | 0.16 | 0.13 |  |  |
| KOREA | 0.20 | 0.18 | 0.19 | 0.20 | | 0.20 | | 0.21 | 0.10 | | 0.21 | | 0.21 | 0.20 | 0.11 |  |  |
| LATVIA | 0.49 | 0.42 | 0.44 | 0.50 | | 0.48 | | 0.50 | 0.51 | | 0.53 | | 0.62 | 0.57 | 0.61 |  |  |
| LEBANON | 0.11 | 0.13 | 0.16 | 0.19 | | 0.22 | | 0.40 | 0.56 | | 0.45 | | 0.21 | 0.23 | 0.33 |  |  |
| LITHUANIA | 0.49 | 0.47 | 0.47 | 0.49 | | 0.51 | | 0.51 | 0.53 | | 0.56 | | 0.52 | 0.52 | 0.50 |  |  |
| LUXEMBOURG | 0.09 | 0.11 | 0.12 | 0.13 | | 0.13 | | 0.14 | 0.16 | | 0.14 | | 0.15 | 0.15 | 0.14 |  |  |
| MALAYSIA | 0.14 | 0.08 | 0.11 | 0.08 | | 0.15 | | 0.17 | 0.16 | | 0.15 | | 0.16 | 0.16 | 0.16 |  |  |
| MEXICO | 0.003 | 0.004 | 0.004 | 0.006 | | 0.007 | | 0.008 | 0.01 | | 0.01 | | 0.01 | 0.007 | 0.01 |  |  |
| MONTENEGRO | 1.16 | 1.14 | 1.04 | 1.29 | | 1.18 | | 1.28 | 1.33 | | 1.22 | | 1.32 | 1.31 | 1.31 |  |  |
| NEW ZEALAND | 3.22 | 3.39 | 3.19 | 3.26 | | 3.21 | | 3.16 | 3.12 | | 3.23 | | 3.01 | 2.94 | 2.99 |  |  |
| NORWAY | 0.61 | 0.59 | 0.58 | 0.60 | | 0.60 | | 0.59 | 0.58 | | 0.57 | | 0.56 | 0.56 | 0.56 |  |  |
| PAKISTAN | 0.02 | 0.02 | 0.04 | 0.03 | | 0.03 | | 0.02 | 0.02 | | 0.03 | | 0.04 | 0.03 | 0.03 |  |  |
| PERU | 0.009 | 0.009 | 0.008 | 0.006 | | 0.006 | | 0.004 | 0.007 | | 0.005 | | 0.005 | 0.004 | 0.007 |  |  |
| PHILIPPINES | 0.05 | 0.05 | 0.06 | 0.06 | | 0.06 | | 0.07 | 0.09 | | 0.08 | | 0.12 | 0.21 | 0.08 |  |  |
| POLAND | 0.50 | 0.50 | 0.60 | 0.60 | | 0.60 | | 0.60 | 0.60 | | 0.60 | | 0.60 | 0.60 | 0.60 |  |  |
| PORTUGAL | 0.34 | 0.35 | 0.37 | 0.38 | | 0.40 | | 0.41 | 0.42 | | 0.40 | | 0.44 | 0.45 | 0.51 |  |  |
| PUERTO RICO | 0.12 | 0.11 | 0.11 | 0.10 | | 0.11 | | 0.10 | 0.10 | | 0.10 | | 0.09 | 0.07 | 0.09 |  |  |
| ROMANIA | 0.22 | 0.25 | 0.23 | 0.24 | | 0.26 | | 0.30 | 0.29 | | 0.31 | | 0.32 | 0.33 | 0.39 |  |  |
| RUSSIA | 0.36 | 0.30 | 0.28 | 0.31 | | 0.30 | | 0.26 | 0.26 | | 0.26 | | 0.24 | 0.25 | 0.25 |  |  |
| SAUDI ARABIA | 0.20 | 0.17 | 0.12 | 0.12 | | 0.20 | | 0.20 | 0.12 | | 0.09 | | 0.20 | 0.18 | 0.19 |  |  |
| SERBIA | 0.77 | 0.93 | 0.91 | 0.92 | | 0.99 | | 1.02 | 1.14 | | 1.03 | | 1.07 | 1.03 | 1.09 |  |  |
| SINGAPORE | 0.13 | 0.12 | 0.14 | 0.15 | | 0.06 | | 0.03 | 0.17 | | 0.05 | | 0.004 | 0.002 | 0.0007 |  |  |
| SLOVAKIA | 0.48 | 0.50 | 0.51 | 0.52 | | 0.52 | | 0.53 | 0.56 | | 0.58 | | 0.59 | 0.59 | 0.62 |  |  |
| SLOVENIA | 0.93 | 0.95 | 0.95 | 0.95 | | 0.98 | | 0.97 | 0.99 | | 0.97 | | 0.95 | 0.98 | 0.99 |  |  |
| SOUTH AFRICA | 0.18 | 0.21 | 0.17 | 0.24 | | 0.35 | | 0.33 | 0.32 | | 0.19 | | 0.19 | 0.21 | 0.25 |  |  |
| SPAIN | 0.33 | 0.36 | 0.38 | 0.39 | | 0.41 | | 0.42 | 0.44 | | 0.45 | | 0.47 | 0.47 | 0.48 |  |  |
| SRI LANKA | 0.02 | 0.02 | 0.01 | 0.01 | | 0.02 | | 0.03 | 0.04 | | 0.05 | | 0.05 | 0.04 | 0.04 |  |  |
| SWEDEN | 0.58 | 0.58 | 0.58 | 0.57 | | 0.57 | | 0.57 | 0.56 | | 0.56 | | 0.56 | 0.55 | 0.56 |  |  |
| SWITZERLAND | 0.91 | 0.88 | 0.86 | 0.85 | | 0.85 | | 0.89 | 0.91 | | 0.88 | | 0.89 | 0.91 | 0.89 |  |  |
| TAIWAN | 0.54 | 0.60 | 0.56 | 0.55 | | 0.58 | | 0.58 | 0.56 | | 0.56 | | 0.56 | 0.55 | 0.57 |  |  |
| THAILAND | 0.30 | 0.37 | 0.46 | 0.37 | | 0.46 | | 0.51 | 0.49 | | 0.43 | | 0.51 | 0.78 | 0.49 |  |  |
| NETHERLANDS | 0.55 | 0.58 | 0.63 | 0.64 | | 0.65 | | 0.65 | 0.66 | | 0.64 | | 0.63 | 0.63 | 0.63 |  |  |
| TUNISIA | 0.07 | 0.07 | 0.06 | 0.09 | | 0.11 | | 0.10 | 0.12 | | 0.13 | | 0.12 | 0.15 | 0.16 |  |  |
| TURKEY | 0.24 | 0.25 | 0.27 | 0.30 | | 0.33 | | 0.30 | 0.31 | | 0.34 | | 0.33 | 0.35 | 0.36 |  |  |
| UAE | 0 | 0.0002 | 0.0002 | 0.0002 | | 0.0002 | | 0.0003 | 0.0004 | | 0.0007 | | 0.0005 | 0.0004 | 0.001 |  |  |
| UK - WHOLE | 0.70 | 0.67 | 0.66 | 0.68 | | 0.66 | | 0.66 | 0.67 | | 0.66 | | 0.66 | 0.67 | 0.71 |  |  |
| UK - SCOTLAND | 0.88 | 0.91 | 0.87 | 0.87 | | 0.87 | | 0.88 | 0.88 | | 0.86 | | 0.84 | 0.80 | 0.80 |  |  |
| UK - WALES | 0.01 | 0.01 | 0.02 | 0.02 | | 0.02 | | 0.02 | 0.02 | | 0.02 | | 0.02 | 0.02 | 0.02 |  |  |
| URUGUAY | 0.21 | 0.26 | 0.25 | 0.28 | | 0.29 | | 0.30 | 0.30 | | 0.35 | | 0.33 | 0.33 | 0.36 |  |  |
| USA | 0.45 | 0.44 | 0.44 | 0.43 | | 0.41 | | 0.35 | 0.34 | | 0.34 | | 0.33 | 0.31 | 0.29 |  |  |
| VENEZUELA | 0.01 | 0.03 | 0.03 | 0.03 | | 0.003 | | 0 | 0 | | 0.0001 | | 0.0004 | 0.01 | 0.02 |  |  |
| VIETNAM | 0.002 | 0.004 | 0.008 | 0.003 | | 0.006 | | 0.008 | 0.007 | | 0.009 | | 0.009 | 0.01 | 0.01 |  |  |

# Table 4 – Comparing rates of clozapine utilisation in 2024 in countries where estimates were available in both IQVIA-MIDAS and national prescribing and administrative databases.

| **Country** | **National administrative and database estimate of clozapine utilisation in 2024** | **IQVIA estimate of clozapine utilisation in 2024** |
| --- | --- | --- |
| Belgium | 0.32 | 0.24 |
| Croatia | 1.10 | 1.21 |
| Estonia | 0.85 | 0.96 |
| Finland | 2.61 | 2.56 |
| Germany | 0.60 | 0.68 |
| Italy | 0.50 | 0.52 |
| Latvia | 0.61 | 0.67 |
| Lithuania | 0.50 | 0.65 |
| Luxembourg | 0.14 | 0.11 |
| Malaysia | 0.16 | 0.24 |
| Netherlands | 0.63 | 0.88 |
| Norway | 0.56 | 0.68 |
| Poland | 0.60 | 0.65 |
| Portugal | 0.51 | 0.59 |
| Slovakia | 0.62 | 0.74 |
| Slovenia | 0.99 | 1.04 |
| Spain | 0.48 | 0.68 |
| Sweden | 0.56 | 0.46 |
| Spearman’s rho = 0.897 (95% CI 0.733, 0.962) ^a,b^, p<0.001.  ^a^ Estimation is based on Fisher's r-to-z transformation.  ^b^ Estimation of standard error is based on the formula proposed by Fieller, Hartley, and Pearson | | |

*Assessment of level of agreement between rates of clozapine utilisation obtained from IQVIA-MIDAS and that obtained from national prescribing and administrative datasets. Author analysis based on IQVIA MIDAS quarterly volume sales data for the period 2014-2024, reflecting estimates of real-world activity. Copyright IQVIA. All rights reserved.*

# Table 5 – Completed STrengthening the Reporting of OBservational studies in Epidemiology (STROBE) Checklist

|  | Item No | Recommendation | Page number |
| --- | --- | --- | --- |
| **Title and abstract** | 1 | (*a*) Indicate the study’s design with a commonly used term in the title or the abstract | 1+2 |
|  |  | (*b*) Provide in the abstract an informative and balanced summary of what was done and what was found | 2 |
| Introduction | | |  |
| Background/rationale | 2 | Explain the scientific background and rationale for the investigation being reported | 5 |
| Objectives | 3 | State specific objectives, including any prespecified hypotheses | 6 |
| Methods | | |  |
| Study design | 4 | Present key elements of study design early in the paper | 6 |
| Setting | 5 | Describe the setting, locations, and relevant dates, including periods of recruitment, exposure, follow-up, and data collection | 6 |
| Participants | 6 | (*a*) *Cohort study*—Give the eligibility criteria, and the sources and methods of selection of participants. Describe methods of follow-up  *Case-control study*—Give the eligibility criteria, and the sources and methods of case ascertainment and control selection. Give the rationale for the choice of cases and controls  *Cross-sectional study*—Give the eligibility criteria, and the sources and methods of selection of participants | n/a |
|  |  | (*b*) *Cohort study*—For matched studies, give matching criteria and number of exposed and unexposed  *Case-control study*—For matched studies, give matching criteria and the number of controls per case | n/a |
| Variables | 7 | Clearly define all outcomes, exposures, predictors, potential confounders, and effect modifiers. Give diagnostic criteria, if applicable | 7+8 |
| Data sources/ measurement | 8* | For each variable of interest, give sources of data and details of methods of assessment (measurement). Describe comparability of assessment methods if there is more than one group | 6 |
| Bias | 9 | Describe any efforts to address potential sources of bias | n/a |
| Study size | 10 | Explain how the study size was arrived at | n/a |
| Quantitative variables | 11 | Explain how quantitative variables were handled in the analyses. If applicable, describe which groupings were chosen and why | 7+8 |
| Statistical methods | 12 | (*a*) Describe all statistical methods, including those used to control for confounding | 7+8 |
|  |  | (*b*) Describe any methods used to examine subgroups and interactions | 7+8 |
|  |  | (*c*) Explain how missing data were addressed | 22–Table 1 explanatory notes |
|  |  | (*d*) *Cohort study*—If applicable, explain how loss to follow-up was addressed  *Case-control study*—If applicable, explain how matching of cases and controls was addressed  *Cross-sectional study*—If applicable, describe analytical methods taking account of sampling strategy | n/a |
|  |  | (*e*) Describe any sensitivity analyses | n/a |

Continued on next page

| Results | | |  |
| --- | --- | --- | --- |
| Participants | 13* | (a) Report numbers of individuals at each stage of study—eg numbers potentially eligible, examined for eligibility, confirmed eligible, included in the study, completing follow-up, and analysed | n/a |
|  |  | (b) Give reasons for non-participation at each stage | n/a |
|  |  | (c) Consider use of a flow diagram | n/a |
| Descriptive data | 14* | (a) Give characteristics of study participants (eg demographic, clinical, social) and information on exposures and potential confounders | n/a |
|  |  | (b) Indicate number of participants with missing data for each variable of interest | n/a |
|  |  | (c) *Cohort study*—Summarise follow-up time (eg, average and total amount) | n/a |
| Outcome data | 15* | *Cohort study*—Report numbers of outcome events or summary measures over time | n/a |
|  |  | *Case-control study—*Report numbers in each exposure category, or summary measures of exposure | n/a |
|  |  | *Cross-sectional study—*Report numbers of outcome events or summary measures | 8 |
| Main results | 16 | (*a*) Give unadjusted estimates and, if applicable, confounder-adjusted estimates and their precision (eg, 95% confidence interval). Make clear which confounders were adjusted for and why they were included | 8-10 |
|  |  | (*b*) Report category boundaries when continuous variables were categorized | n/a |
|  |  | (*c*) If relevant, consider translating estimates of relative risk into absolute risk for a meaningful time period | n/a |
| Other analyses | 17 | Report other analyses done—eg analyses of subgroups and interactions, and sensitivity analyses | 9+10 |
| Discussion | | |  |
| Key results | 18 | Summarise key results with reference to study objectives | 10 |
| Limitations | 19 | Discuss limitations of the study, taking into account sources of potential bias or imprecision. Discuss both direction and magnitude of any potential bias | 12 |
| Interpretation | 20 | Give a cautious overall interpretation of results considering objectives, limitations, multiplicity of analyses, results from similar studies, and other relevant evidence | 12 |
| Generalisability | 21 | Discuss the generalisability (external validity) of the study results | 12 |
| Other information | | |  |
| Funding | 22 | Give the source of funding and the role of the funders for the present study and, if applicable, for the original study on which the present article is based | 8 |

*Give information separately for cases and controls in case-control studies and, if applicable, for exposed and unexposed groups in cohort and cross-sectional studies.

# Table 6 – Details of correlation and regression analyses of factors associated with estimates of clozapine utilisation in 2024

1. **Descriptive summary of dependent variables**

| **Psych_100,000 (number of psychiatrists per 100,000 population)**  Valid (n=60)  Missing (n=15) | **Stringency Index**  Valid (n=60)  Missing (n=15) |
| --- | --- |
| Mean = 12.6242 | Mean = 65.0500 |
| Median = 11.700 | Median = 79.5000 |
| Standard Deviation = 9.77018 | Standard Deviation = 23.41478 |
| Minimum = 1.00 | Minimum = 6.00 |
| Maximum = 53.00 | Maximum = 100.0 |

1. **Spearman’s rank correlation coefficient – Psychiatrists/100,000 population/Clozapine utilisation in 2024**

| Psych_100000 - DDD_1000_2024 | Spearman’s rho = 0.596 | Significance(2-tailed) = <0.001 | 95% confidence interval (lower) = 0.397 | 95% confidence interval (upper) =0.742 |
| --- | --- | --- | --- | --- |

1. **Spearman’s rank correlation coefficient – Haematological Stringency Index/Clozapine utilisation in 2024**

| Stringency_Index - DDD_1000_2024 | Spearman’s rho =0.427 | Significance (2-tailed) = <0.001 | 95% confidence interval (lower) = 0.185 | 95% confidence interval (upper) =0.621 |
| --- | --- | --- | --- | --- |

1. **Regression analysis**

| **Model summary**  Predictors: (Constant), Psych_100000, Stringency_Index  Dependent Variable: Ln_DDD_1000_day | | | |
| --- | --- | --- | --- |
| R=0.588 | R^2^  =0.359 | Adjusted R^2^ =0.332 | Standard Error of the Estimate = 1.439 |

| **Coefficients** | | | | | | | |
| --- | --- | --- | --- | --- | --- | --- | --- |
|  | **Unstandardized B** | **Standard Error** | **Standardized Coefficients Beta** | **t** | **Significant (2-tailed)** | **95% confidence interval (lower)** | **95% confidence interval (upper)** |
| Constant | -3.297 | 626 |  | -5.270 | <.001 | -4.555 | -2.039 |
| Stringency_Index | .007 | .011 | .096 | .651 | .518 | -.016 | .030 |
| Psych_100000 | .112 | .031 | .534 | 3.621 | <.001 | .050 | .174 |

# Plot 1 - Plot of individual countries’ clozapine utilisation in 2024 by number of psychiatrists/100,000 population


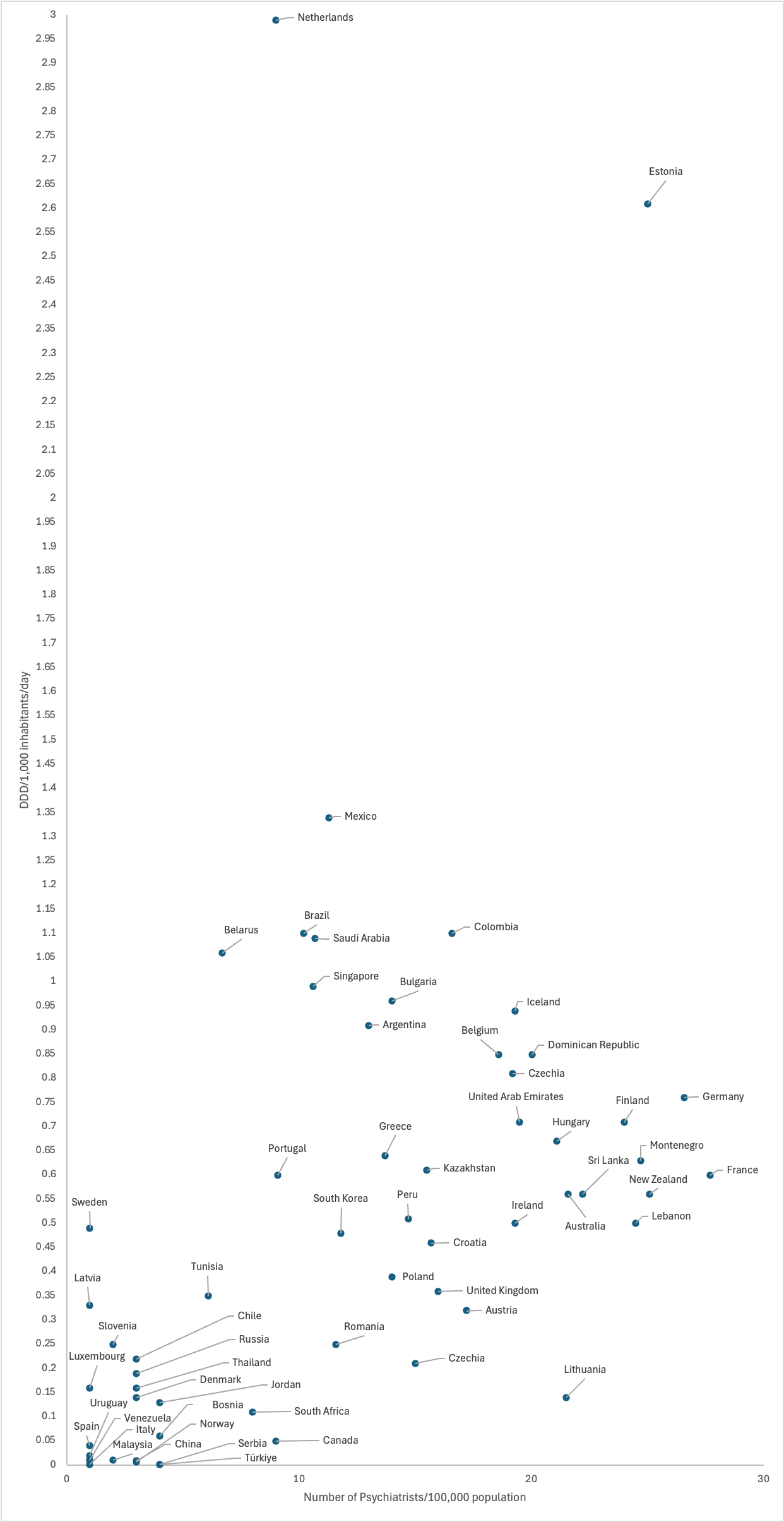


*Author analysis based on IQVIA MIDAS quarterly volume sales data for the period 2014-2024, reflecting estimates of real-world activity. Copyright IQVIA. All rights reserved.*

# Haematological Monitoring Stringency Index – Details of Eloyede et al method

According to this method a figure of 0-100 has been assigned to individual countries following consideration of:

1. Haematological monitoring parameters
2. Monitoring frequency
3. Thresholds for clozapine discontinuation according to:
   1. Absolute neutrophil counts
   2. White cell count
4. Rechallenge restrictions
5. The presence of modified monitoring criteria to account for benign ethnic neutropenia.

**Reference paper with assigned haematological Stringency Indices**: Oloyede E, Blackman G, Whiskey E, Bachmann C, Dzahini O, Shergill S, Taylor D, McGuire P, MacCabe J. Clozapine haematological monitoring for neutropenia: a global perspective. *Epidemiol Psychiatr Sci*. 2022;31:e83.
